# Supplementary material for: Physical characteristics of soil-biodegradable and nonbiodegradable plastic mulches impact conidial splash dispersal of Botrytis cinerea
Source: PLoS One. 2023 May 8;18(5):e0285094. doi: 10.1371/journal.pone.0285094 (PMC10166481; doi:10.1371/journal.pone.0285094)
Supplement: S1 Table — (DOCX) [file pone.0285094.s001.docx]

**S1 Table. Mulch tension measurements in a mulched ‘Albion’ strawberry field in Mount Vernon, WA.**

| Mulch^a^ | Tension (N)^b^ | Position^c^ |
| --- | --- | --- |
| PE | 0.76 | Edge |
| PE | 0.73 | Center |
| PE | 0.74 | Middle |
| PE | 0.75 | Middle |
| PE | 0.74 | Edge |
| BDM | 0.74 | Edge |
| BDM | 0.76 | Middle |
| BDM | 0.73 | Edge |
| BDM | 0.74 | Middle |
| BDM | 0.78 | Center |
| Weedmat | 0.73 | Edge |
| Weedmat | 0.75 | Middle |
| Weedmat | 0.72 | Center |
| Weedmat | 0.78 | Middle |
| Weedmat | 0.72 | Edge |

^a^ Mulch measurements were taken at random locations on polyethylene (PE) mulch, embossed soil-biodegradable mulch (BDM) and weedmat (a woven fabric PE mulch) raised beds.

^b^ Mulch tension was measured using a tension meter (Chatillon 516 Series linear push/pull scale, Ametek, Berwyn, PA, USA).

^c^ Data collection points were made at the ‘edge’ and ‘middle’ of the raised beds; measurements were also collected near the planting holes noted as the ‘center’ position.
