# Supplementary material for: Physical characteristics of soil-biodegradable and nonbiodegradable plastic mulches impact conidial splash dispersal of Botrytis cinerea
Source: PLoS One. 2023 May 8;18(5):e0285094. doi: 10.1371/journal.pone.0285094 (PMC10166481; doi:10.1371/journal.pone.0285094)
Supplement: S2 Table — (DOCX) [file pone.0285094.s002.docx]

**S2 Table. Uniformity tests for standardizing rain simulator system before running splash dispersal experiments.**

| Petri plates arrangement direction | Distance from inoculum source (cm) | Accumulated water volume (ml)^a^ |
| --- | --- | --- |
| South | 10 | 0.9±0.04 |
| South | 16 | 1.2±0.06 |
| South | 22 | 1.8±0.08 |
| South | 28 | 2.7±0.13 |
| South | 34 | 3.4±0.23 |
| East | 10 | 0.8±0.05 |
| East | 16 | 1.0±0.06 |
| East | 22 | 1.1±0.08 |
| East | 28 | 1.2±0.05 |
| East | 34 | 1.2±0.04 |
| Southeast | 10 | 1.0±0.07 |
| Southeast | 16 | 1.3±0.05 |
| Southeast | 22 | 1.6±0.02 |
| Southeast | 28 | 1.7±0.11 |
| Southeast | 34 | 2.0±0.15 |

^a^All three nozzles on the rain simulator system were adjusted to a 210˚ arc and a low flow rate (68 liter/hour). The rain simulator system was set to run 2 minutes. Empty Petri dishes (6 cm diameter) were placed at 10, 16, 22, 28, 34 cm distance from the inoculum pool. Uniformity tests were conducted three times in south, east and southeast directions. Data were averaging across three replicates and standard errors were calculated.
