# Supplementary material for: Physical characteristics of soil-biodegradable and nonbiodegradable plastic mulches impact conidial splash dispersal of Botrytis cinerea
Source: PLoS One. 2023 May 8;18(5):e0285094. doi: 10.1371/journal.pone.0285094 (PMC10166481; doi:10.1371/journal.pone.0285094)
Supplement: S3 Table — (DOCX) [file pone.0285094.s003.docx]

**S3 Table. Assessment of conidial germination rates prior to running the splash dispersal experiment.**

| Conidial suspension concentration | Germination rate^a^ |
| --- | --- |
| 2×10^6^/ml | 100% |
| 2.5×10^6^/ml | 100% |
| 3×10^6^/ml | 98% |

^a^ *Botrytis cinerea* conidial suspension was plated on Botrytis spore trap media (BSTM) and incubated at 22 ℃ for 18 hours in light. After 18 hours, 50 conidia per each concentration were observed using a compound microscope at 40X magnification. A conidium was considered germinated if germ tube length was around half width of conidium. Observations were made on different days at different conidial suspension concentrations.
