# Supplementary material for: Physical characteristics of soil-biodegradable and nonbiodegradable plastic mulches impact conidial splash dispersal of Botrytis cinerea
Source: PLoS One. 2023 May 8;18(5):e0285094. doi: 10.1371/journal.pone.0285094 (PMC10166481; doi:10.1371/journal.pone.0285094)
Supplement: S4 Table — (DOCX) [file pone.0285094.s004.docx]

**S4 Table. Germination rate of all dispersed *Botrytis cinerea* conidia^a^ recovered from the rain splash dispersal experiment.**

| Mulch^b^ | Dispersal distance from inoculum source (cm) | Germination rate (%)^c^ |
| --- | --- | --- |
| BDM | 10 | 82.8 |
| BDM | 16 | 58.8 |
| BDM | 22 | 39.1 |
| BDM | 28 | 78.3 |
| BDM | 34 | 20.0 |
| PE | 10 | 79.5 |
| PE | 16 | 56.3 |
| PE | 22 | 47.6 |
| PE | 28 | . ^b^ |
| PE | 34 | . |
| Weedmat | 10 | 79.4 |
| Weedmat | 16 | 47.1 |
| Weedmat | 22 | 44.4 |
| Weedmat | 28 | 70.6 |
| Weedmat | 34 | . |

^a^ *Botrytis cinerea* conidial suspension was plated on Botrytis spore trap media (BSTM) and incubated at 22 ℃ for 18 hours in light. After 18 hours, 50 conidia per each concentration were observed using a compound microscope at 40X magnification. A conidium was considered germinated if germ tube length was around half width of conidium. Data were averaged across five replications.

^b^ Mulch treatments include PE mulch 25.5 μm (Filmtech, LLC., Stanley, WI, USA); (2) weedmat 85 g/m^2^ (Extenday, Union Gap, WA, USA); (3) BDM 25.5 μm (Organix Solutions, Bloomington, MN, USA).

^c^ “.” means there were not sufficient conidia to evaluate germination.
